# The Pathogenesis of Periodic Fever, Aphthous Stomatitis, Pharyngitis, and Cervical Adenitis Syndrome: A Review of Current Research

**DOI:** 10.1155/2015/563876

**Published:** 2015-09-17

**Authors:** Barbara Kraszewska-Głomba, Agnieszka Matkowska-Kocjan, Leszek Szenborn

**Affiliations:** Department and Clinic of Pediatric Infectious Diseases, Wroclaw Medical University, 2-2A Chałubińskiego, 50-368 Wroclaw, Poland

## Abstract

*Background*. PFAPA syndrome is a chronic disease that is characterized by recurrent episodes of high fever, aphthous stomatitis, pharyngitis, and cervical adenitis. Knowledge regarding the etiology of PFAPA is limited. *Objectives*. To provide up-to-date information considering etiology of PFAPA syndrome, by summarizing what has been explored and established in this area so far. *Materials and Methods*. PubMed, Web of Science, and Scopus databases were searched for pertinent reports. Eventually 19 articles were selected. The results were classified into categories regarding three areas of interest: familial occurrence, genetic basis, and immunological mechanisms of PFAPA. *Results*. Recent findings suggest that there is a familial tendency to PFAPA but the level of evidence does not warrant definite conclusions. The absence of a clear monogenic trait indicates a heterogenous, polygenic, or complex inheritance of PFAPA syndrome. As two mutations with a possible functional effect on the inflammasomes (MEFV E148Q and NLRP3 Q703K) have been found in several PFAPA cohorts, the role of inflammasome-related genes in PFAPA pathogenesis cannot be excluded. Immunological mechanisms of PFAPA involve an abnormal, IL-1*β* dependent innate immune response to an environmental trigger, which leads to Th1-driven inflammation expressed by recruitment of T-cells to the periphery.

## 1. Introduction

Periodic fever, aphthous stomatitis, pharyngitis, and cervical adenitis (PFAPA) [1] syndrome belongs to the spectrum of autoinflammatory diseases characterized by spontaneous episodes of inflammation which are not accompanied by the usual hallmarks of autoimmunity—high-titer autoantibodies or autoreactive T-cells [1]. In terms of symptomatology PFAPA is considered one of the periodic fever syndromes, but in regard of etiology it is categorized, along with Behçet's disease and sJIA (Systemic-onset Juvenile Idiopathic Arthritis), as idiopathic disease [1]. Many possible causative factors have been explored so far including infectious agents, immunological mechanisms, and genetic predisposition. Rare occurrence of PFAPA in adults [2] and slight male predominance [3–6] absence of known autoreactive antibodies or T-cells [1, 7], as well as shortening of intervals between febrile attacks after corticosteroid-treatment [3, 4], speak highly against autoimmune-mediated disease [8]. Rapid response to steroids [3–6] speaks against infection and for dysregulated inflammatory cytokine production [9]. While the occurrence of the disease in different geographical regions [8], among various ethnicities [10], might argue for an infectious vector with worldwide prevalence, studies so far have failed to identify any microbiological agent (or agents) potentially involved in pathogenesis of PFAPA [3, 11, 12]. Due to the lack of evidence of autoimmune or infectious cause, PFAPA has been considered an autoinflammatory disease, but the exact pathogenesis or genetic background remains unclear [9, 13–15]. The purpose of this review is to provide up-to-date information considering etiology of PFAPA syndrome, by summarizing what has been explored and established in this area so far.

## 2. Materials and Methods

PubMed, Web of Science, and Scopus databases were searched for pertinent reports; the last search update was performed on June 07, 2015. The terms “PFAPA” and “etiology or pathogenesis or pathophysiology” were used as the keywords. No language restrictions nor a date range was set. Some publications were identified by screening references of retrieved articles. After removing duplicates, 96 records were identified. Two authors (BK-G, AMK) independently screened all reports by title or abstract for eligibility. The authors excluded comments, letters, reviews, and papers that did not study or report our outcomes of interest. If either of the reviewers considered the abstract potentially relevant, full-text articles were retrieved and then evaluated by both reviewers for their suitability for inclusion. Discrepancies were resolved by group discussion. Eventually 19 publications were selected. The following information was extracted from each study: the first author's name, language, country, year of publication and patient's characteristics (including ethnicity and age), definition and numbers of cases and controls, laboratory methods, and outcomes.

The results were classified into three categories regarding three areas of interest: familial occurrence, genetic basis, and immunological mechanisms of PFAPA.

## 3. Results and Discussion

### 3.1. Is PFAPA a Sporadic Disease?

Contrary to the majority of periodic fever syndromes, which are hereditary monogenic disorders, genetic basis has not yet been established for PFAPA, and for a long time it was regarded as a sporadic disease [1, 4, 8, 16]. Familial occurrence was not mentioned in the reported series of PFAPA patients [2–6]. However, within the last few years several cases of siblings [17–21] including a case of siblings and their mother have been reported [19]. In 2010 Cochard et al. [21] published data questioning the nonhereditary nature of PFAPA. They interviewed 84 PFAPA patients from 8 different countries and found positive family history for recurrent fever in 38 (45%) of them. In 76% (29/38) of the cases, the affected family member was a sibling or a parent. The recurrent fever met PFAPA criteria in 12% (10/84) of the reported cases. All 47 healthy children in the control group had negative family history of recurrent fever. The limitations of the study are the following: a retrospective character of the study, a relatively small control group, potential mistakes deriving from relying on the respondent's subjective knowledge and possibly an incomplete record of the diseases present in the family members. In another large comparative study (130 PFAPA patients, mostly Italian) positive family history was found only in 13.8% of cases [22]. On the other hand, the result was similar in PFAPA and hereditary recurrent fever syndromes such as FMF and MKD (13.8%, 14.3%, and 9.1%, resp.). Nevertheless, comparing to PFAPA, the other two diseases were significantly underrepresented (FMF—7, MKD—33 patients). Different results of these two studies may be associated with methodology. The Italian study was conducted prospectively on a bigger study population. Additionally, in the Italian study genetic testing was performed in all PFAPA patients to exclude hereditary periodic fever syndromes, while in Cochard's study monogenic autoinflammatory diseases were excluded mainly by clinical presentation (genetic testing only in 20/84 patients). In a lately reported single-center longitudinal study, positive family history (recurrent fever and/or tonsillitis) was found in 78% (50/64) of PFAPA patients, which is the highest rate ever reported [20]. However, a recent analysis of a large international web-based multicentre cohort showed lower (26.9%, 81/301) prevalence of positive family history in PFAPA patients [23].

In summary, recent findings strongly suggest that there is a familial tendency to PFAPA but the level of evidence does not warrant definite conclusions.

### 3.2. What Is the Genetic Background of PFAPA Syndrome?

Reports suggesting familial susceptibility to PFAPA reinforced the search of its genetic background. Recently Bens et al. identified SPAG7 as a candidate gene for PFAPA [24]. Nevertheless mutation of the gene was reported only in 1 patient with PFAPA syndrome and the significance of this finding is still to be established. In the most recent report by Di Gioia et al. exome sequencing of 11 PFAPA patients did not reveal any unique mutation that would be present in all the affected individuals, indicating that PFAPA is not a monogenic condition [13]. Since the clinical features of PFAPA and hereditary periodic fever syndromes (PFSs) are often overlapping [22], PFAPA cohorts have been searched for the presence of mutations that are known to cause other PFSs.

Padeh et al. were the first to report heterozygous variants of MEFV (a gene associated with familial Mediterranean fever) in patients with PFAPA [4]; however all of those patients belonged to ethnicities with high frequency of carriers [4, 8]. Dagan et al. found MEFV variants (M694V, V726A, or E148Q) in 28.1% (16/57) of PFAPA patients from Israel [25]. There was no control group, but the result is comparable to that of the general population in Israel (30–40%) [26, 27]. In a big prospective Israeli study by Berkun et al., MEFV variants (mostly M694V) were identified in as much as 55.4% (65/124) of children with PFAPA [7]. However, according to the authors, due to a selection of bias reasons, the study should not be used to assess the prevalence of heterozygous MEFV mutations in PFAPA patients [7]. Japanese authors (Taniuchi et al.) reported frequency of MEFV variants in PFAPA patients of 65% (13/20) [28], which is higher than that in the aforementioned Israeli studies [4, 7, 25]. This might be due to differences in number of the included patients, their ethnicity, and selection method. In comparison to the control group (62 healthy volunteers) the incidence of two MEFV variants (E148Q-L110P and P369S-R408Q) was significantly higher in PFAPA patients. Three of those variants (E148Q, P369S, and R408Q) along with three others (A289V, I591T, and K695R) were also found in 8% (5/62) of children with PFAPA in a recent report from Slovenia [20]. In a comprehensive genetic study by Di Gioia et al., among 68 individuals from 14 families affected by PFAPA, E148Q variant was identified in two family members [13]. The role of E148Q variant is controversial, but it might have a functional effect on inflammasomes, macromolecular IL-1*β*-activating complexes, which have recently been associated with PFAPA pathophysiology [9, 13–15, 28]. P369S and K695R are low-penetrant variants [29, 30] and the function of the other variants remains unknown [20].

In both Taniuchi's and Berkun's study, shorter duration of PFAPA febrile attacks was found in patients with MEFV mutations, compared to those without them [7, 28]. Berkun et al. also observed lower rates of regular cyclic patterns and oral aphthae among the MEFV carriers (especially M694V) [7].

In a Swiss study from 2013 variants of NLRP3—a gene involved in CAPS pathogenesis—were found in as much as 23% (12/57) of PFAPA patients [15], which is a higher proportion than that in the general population [31, 32]. Nevertheless, a statistical analysis of single NLRP3 variants (V198M, R488K, and Q703K) proved significant difference for only one mutation (R488K) [15] of limited significance [32]. V198M variant, which is often associated with milder forms of CAPS [33], was recently shown not to cosegregate with PFAPA [13]. The Q703K variant was recently found in 14.5% (9/62) of PFAPA patients as well as in 12% (12/100) healthy subjects from Slovenia (statistical significance has not been reached) [20] and in one of the 14 families studied by Di Gioia et al. [13]. This polymorphism has been recognized as a gain-of-function alteration leading to an overactive NLRP3 inflammasome and might play a role in PFAPA pathogenesis [34].

The hitherto published data does not support the involvement of mutations associated with TRAPS (gene TNFRSF1A) or MVD (gene MVK) in PFAPA etiology [13, 15, 20, 25, 28]. A mild low-penetrance, rather asymptomatic variant of TNFRSF1A (R92Q) was found in 1 out of 50 Israeli patients [25] and in 1 out of 57 cases from Switzerland [15]. In a recent study by Di Gioia et al. a rare mutation, that is usually associated with a mild form of TRAPS (R121Q), was found in one of the families affected with PFAPA [13]. Only one case of MVK mutation in a patient with PFAPA has been reported [15].

Recently published results of an exhaustive screening for mutation in genes responsible for hereditary recurrent fever syndromes [13] indicate that their involvement in PFAPA etiology is very unlikely. Only a few rare variants were found, but their frequency in PFAPA patients was similar to those observed in general population and some of these changes did not cosegregate with the phenotype [13].

In summary, the absence of a clear monogenic trait indicates a heterogenous, polygenic, or complex inheritance of PFAPA syndrome. Currently, there is no strong evidence to support the association between genes confirmative of hereditary PEF's and PFAPA etiology. TNFRSF1A or MVK mutations were sporadically reported in patients with PFAPA and do not seem to be involved in the disease. Most of the identified NLRP3 and MEFV variants are low-penetrance mutations or benign polymorphisms and their relevance to PFAPA pathogenesis is uncertain. As two mutations with a possible functional effect on the inflammasomes (MEFV E148Q and NLRP3 Q703K) have been found in several PFAPA cohorts, the role of inflammasome-related genes in PFAPA pathogenesis cannot be excluded.

### 3.3. What Immunologic Mechanisms Are Involved in PFAPA Flares?

It is not improbable that MEFV and/or NLRP3 variants are involved in the pathogenesis of PFAPA, since they are functionally linked to inflammatory reactions taking place during PFAPA flares. Protein products of both of these genes regulate activation of caspase-1, which processes proIL-1*β* and proIL-18 to mature forms [9, 35]. IL-1*β* and IL-18 are important mediators of the inflammatory response leading to fever, elevated acute-phase proteins, and neutrophilia [1]. It has been associated with the pathogenesis of the majority of hereditary periodic fever syndromes and recently also with PFAPA syndrome, [1, 9, 14, 15, 36–40].

Stojanov et al. showed that fever attacks in 6 PFAPA patients led to significant increase in serum concentrations of IL-1*β* and other proinflammatory cytokines (IL-6, IFN*γ*, and TNF-*α*) when compared to healthy controls (11 children) [40]. Brown et al. also reported increases in IL-6 sera concentrations during PFAPA flares, whereas levels of IL-1*β*, TNF-*α*, and IFN*γ* remain low [14]. Similar results were presented in a Norwegian study comparing 22 PFAPA patients and 14 children with pneumonia [41]. As the time after the onset of fever, in which sera were drawn, was the shortest in Stojanov et al.'s study (6–12 hours) [40], it was presumed that IL-1*β* and TNF-*α* peak early in the fever period and then quickly approach homeostatic levels [41]. This was not confirmed by Kubota et al., who found increased levels of IL-1*β* and TNF-*α* in blood collected from 9 PFAPA patients within 96 hours from fever onset [36]. In a recent study among 33 patients from Japan IL-1*β* and TNF-*α* were not elevated, while IL-18, IL-6, and IFN*γ* increased during PFAPA febrile attacks [42]. Two other studies failed to find elevated levels of IL-1*β* in PFAPA patients; nevertheless, indirect evidence of the increased IL-1*β* production during PFAPA fever attacks was discovered [9, 15]. Stojanov et al. reported overexpression of IL-1-related and inflammasome-related genes, increased sera concentrations of IL-1*β*-induced proinflammatory cytokines (IL-6 and G-CSF), and, finally, prompt clinical response to IL-1 receptor antagonist treatment (anakinra) in 5/5 PFAPA patients [9]. Kolly et al. found increased levels of caspase-1 and significantly higher secretion of IL-1*β* by stimulated peripheral blood mononuclear cells during febrile episodes compared to afebrile periods [15].

Increase in serum concentrations of IL-18 was demonstrated in several studies [9, 36, 42]. IL-18 stimulates release of IFN*γ* that was also elevated in the serum of PFAPA patients [9, 36, 40, 42] as well as IFN*γ* inducible chemokines [9, 15, 40].

Elevated levels of IL-1*β*, IL-18, IL-6, and IFN*γ* point to innate immunity dysregulation as the key pathomechanism of PFAPA attacks. The concept is advocated by other immunological aberrations during PFAPA flares such as monocytosis, increased count [9, 14, 15, 41, 43] and characteristic features of neutrophils [40], increased secretion of proinflammatory chemokines (IP10/CXCL10, MIG/CXCL9) [9, 14, 41], and upregulated transcription of complement genes [9].

Increased neutrophil and monocyte counts during febrile episodes have been reported in several PFAPA cohorts [9, 14, 15, 41, 43]. Furthermore, Stojanov et al. found significant alteration of neutrophil functions during PFAPA flares: diminished rates of spontaneous apoptosis, increased generation of intracellular NADPH oxidase-derived ROS (Reactive Oxygen Species), and signatures of priming such as granule mobilization and receptor upregulation of the cell surface [40].

Chemokines are induced directly by early innate response mechanisms and under the influence of IFN*γ* [41]. They are strong chemoattractants of T-cells to the inflammation sites and may act as a link between innate and adaptive immune responses [9, 14, 41], which in PFAPA is mainly Th1-driven [9, 41]. Th1-induced elevation of IFN*γ* and CXCL10 levels [9, 14, 15, 41] in the absence of the increased Th2- and Th17-cytokines [14] weighs heavily in favour of Th1-type inflammatory response in PFAPA. The suppression of Th2 type response is supported by low IL-4 levels and low expression of IL-4 gene in peripheral blood [40] and in the tonsils [44]. Additionally, lack of eosinopenia has been linked to Th2-driven inflammation [45, 46], whereas several studies reported eosinopenia in PFAPA patients during febrile episodes [9, 14, 41, 43], which also argues in favour of Th1-driven immune response in PFAPA syndrome.

Involvement of adaptive immunity in PFAPA pathogenesis is indicated by fluctuations in T lymphocytes and increase in serum IFN*γ* levels during the attack periods [9, 14, 15]. A decrease in the number of circulating lymphocytes during PFAPA flares was found in several studies [9, 14, 15, 41], two of which reported a decrease in both CD4+ and CD8+ lymphocyte counts [9, 41]. As previously suggested, this may result from activation and recruitment of T-cells to peripheral tissues and is clinically reflected by tonsillitis and cervical adenitis [9, 41, 42, 47]. Two recently published studies support this hypothesis. Yamazaki et al. showed, that neutrophils and monocytes of PFAPA patients (*n* = 33) during attacks strongly expressed CD64, an Fc*γ* receptor, which might be upregulated by IFN*γ*, possibly from retention of activated T-cells in the periphery [42]. A study by Petra et al. revealed increased levels of CD8+ T-cells, the transitional B cells, and naive stages of both the CD4+ and CD8+ T-cells in the tonsils from PFAPA patients (*n* = 10), compared to tonsils from children with obstructive sleep apnea syndrome (*n* = 10) [47].

The studies exploring pathophysiology of PFAPA [9, 14, 15, 36, 40–43, 47] differed in terms of characteristics and size of the PFAPA cohort, presence and type of control group, examined proteins, and laboratory methods. Most of them support the hypothesis of an abnormal, IL-1*β* dependent innate immune response to an environmental trigger that leads to Th1-driven inflammation, expressed by recruitment of T-cells to periphery.

In summary, PFAPA is an autoinflammatory disease of compound, heterogeneous etiology. It shares some clinical and pathogenic features with monogenic recurrent fever syndromes; however, studies have failed to identify its genetic basis. Possible involvement of MEFV and NLRP mutations is in line with their functional connection to IL-1*β* dependent innate inflammatory response, which might be involved in PFAPA pathogenesis. Most autoinflammatory diseases derive from genetic variants of the innate immune system, and except for TRAPS, all monogenic periodic syndromes belong to IL-1*β*-regulated autoinflammatory diseases [1]. However, the pathophysiology of PFAPA syndrome seems to be more complex. Inflammatory response in PFAPA is also driven by Th1-type adaptive immune response, which also dominates in several autoimmune diseases, for example, Hashimoto's thyroiditis, Grave's disease, Crohn's disease, psoriasis, Type 1 diabetes, and Rheumatoid arthritis [48]. Th1-driven immune response mainly develops following infections by viruses, intracellular bacteria, and parasites [49], while IL-1*β* activation has been shown to be involved in bacterial, viral, and fungal infections [50]. The role of a viral or other infectious agents in PFAPA etiology is unknown, though the hitherto published studies are pointing to collaboration of environmental and immunological factors in a genetically inclined individual.

## 4. Conclusions

Despite recent advances in unravelling the molecular aspects of PFAPA pathogenesis, there still seem to be more questions than answers. Areas for further research include identifying genes and microbiological factors involved in PFAPA etiology, as well as the consequent exploration of immunological mechanisms that could improve diagnostic and treatment regimens for PFAPA patients in the future.

## References

[1] Masters S. L., Simon A., Aksentijevich I., Kastner D. L. (2009). Horror autoinflammaticus: the molecular pathophysiology of autoinflammatory disease. *Annual Review of Immunology*.

[2] Padeh S., Stoffman N., Berkun Y. (2008). Periodic fever accompanied by aphthous stomatitis, pharyngitis and cervical adenitis syndrome (PFAPA syndrome) in adults. *Israel Medical Association Journal*.

[3] Thomas K. T., Feder H. M., Lawton A. R., Edwards K. M. (1999). Periodic fever syndrome in children. *The Journal of Pediatrics*.

[4] Padeh S., Brezniak N., Zemer D. (1999). Periodic fever, aphthous stomatitis, pharyngitis, and adenopathy syndrome: clinical characteristics and outcome. *Journal of Pediatrics*.

[5] Tasher D., Somekh E., Dalal I. (2006). PFAPA syndrome: new clinical aspects disclosed. *Archives of Disease in Childhood*.

[6] Feder H. M., Salazar J. C. (2010). A clinical review of 105 patients with PFAPA (a periodic fever syndrome). *Acta Paediatrica*.

[7] Berkun Y., Levy R., Hurwitz A. (2011). The familial Mediterranean fever gene as a modifier of periodic fever, aphthous stomatitis, pharyngitis, and adenopathy syndrome. *Seminars in Arthritis and Rheumatism*.

[8] Long S. S. (1999). Syndrome of periodic fever, aphthous stomatitis, pharyngitis and adenitis (PFAPA)—what it isn’t what is it?. *Journal of Pediatrics*.

[9] Stojanov S., Lapidus S., Chitkara P. (2011). Periodic fever, aphthous stomatitis, pharyngitis, and adenitis (PFAPA) is a disorder of innate immunity and Th1 activation responsive to IL-1 blockade. *Proceedings of the National Academy of Sciences of the United States of America*.

[10] Lierl M. (2007). Periodic fever syndromes: a diagnostic challenge for the allergist. *Allergy*.

[11] Marshall G. S., Edwards K. M., Butler J., Lawton A. R. (1987). Syndrome of periodic fever, pharyngitis, and aphthous stomatitis. *The Journal of Pediatrics*.

[12] Freeman S., Bhatt A., Pedamallu C. (2014). A121: in search of infectious triggers of periodic Fever, aphthous stomatitis, pharyngitis and adenitis syndrome. *Arthritis & Rheumatology*.

[13] Di Gioia S. A., Bedoni N., von Scheven-Gête A. (2015). Analysis of the genetic basis of periodic fever with aphthous stomatitis, pharyngitis, and cervical adenitis (PFAPA) syndrome. *Scientific Reports*.

[14] Brown K. L., Wekell P., Osla V. (2010). Profile of blood cells and inflammatory mediators in periodic fever, aphthous stomatitis, pharyngitis and adenitis (PFAPA) syndrome. *BMC Pediatrics*.

[15] Kolly L., Busso N., von Scheven-Gete A. (2013). Periodic fever, aphthous stomatitis, pharyngitis, cervical adenitis syndrome is linked to dysregulated monocyte IL-1beta production. *Journal of Allergy and Clinical Immunology*.

[16] Cazeneuve C., Geneviève D., Amselem S., Hentgen V., Hau I., Reinert P. (2003). MEFV gene analysis in PFAPA. *Journal of Pediatrics*.

[17] Sampaio I. C. R. M., Rodrigo M. J., Monteiro Marques J. G. D. P. (2009). Two siblings with periodic fever, aphthous stomatitis, pharyngitis, adenitis (Pfapa) syndrome. *Pediatric Infectious Disease Journal*.

[18] Valenzuela P. M., Majerson D., Tapia J. L., Talesnik E. (2009). Syndrome of periodic fever, aphthous stomatitis, pharyngitis, and adenitis (PFAPA) in siblings. *Clinical Rheumatology*.

[19] Adachi M., Watanabe A., Nishiyama A. (2011). Familial cases of periodic fever with aphthous stomatitis, pharyngitis, and cervical adenitis syndrome. *The Journal of Pediatrics*.

[20] Perko D., Debeljak M., Toplak N., Avčin T. (2015). Clinical features and genetic background of the periodic Fever syndrome with aphthous stomatitis, pharyngitis, and adenitis: a single center longitudinal study of 81 patients. *Mediators of Inflammation*.

[21] Cochard M., Clet J., Le L. (2010). PFAPA syndrome is not a sporadic disease. *Rheumatology*.

[22] Gattorno M., Caorsi R., Meini A. (2009). Differentiating PFAPA syndrome from monogenic periodic fevers. *Pediatrics*.

[23] Hofer M., Pillet P., Cochard M.-M. (2014). International periodic fever, aphthous stomatitis, pharyngitis, cervical adenitis syndrome cohort: Description of distinct phenotypes in 301 patients. *Rheumatology*.

[24] Bens S., Zichner T., Stütz A. M. (2014). SPAG7 is a candidate gene for the periodic fever, aphthous stomatitis, pharyngitis and adenopathy (PFAPA) syndrome. *Genes and Immunity*.

[25] Dagan E., Gershoni-Baruch R., Khatib I., Mori A., Brik R. (2010). MEFV, TNF1rA, CARD15 and NLRP3 mutation analysis in PFAPA. *Rheumatology International*.

[26] Kogan A., Shinar Y., Lidar M. (2001). Common MEFV mutations among Jewish ethnic groups in Israel: high frequency of carrier and phenotype III states and absence of a perceptible biological advantage for the carrier state. *American Journal of Medical Genetics*.

[27] Stoffman N., Magal N., Shohat T. (2000). Higher than expected carrier rates for familial Mediterranean fever in various Jewish ethnic groups. *European Journal of Human Genetics*.

[28] Taniuchi S., Nishikomori R., Iharada A., Tuji S., Heike T., Kaneko K. (2013). MEFV variants in patients with PFAPA syndrome in Japan. *The Open Rheumatology Journal*.

[29] Topaloglu R., Yildiz C., Taskiran E. (2013). PW01-004—the sequence analysis in E148Q homozygous patients. *Pediatric Rheumatology*.

[30] Aksentijevich I., Torosyan Y., Samuels J. (1999). Mutation and haplotype studies of familial Mediterranean fever reveal new ancestral relationships and evidence for a high carrier frequency with reduced penetrance in the Ashkenazi Jewish population. *American Journal of Human Genetics*.

[31] Neven B., Callebaut I., Prieur A.-M. (2004). Molecular basis of the spectral expression of *CIAS1* mutations associated with phagocytic cell-mediated autoinflammatory disorders CINCA/NOMID, MWS, and FCU. *Blood*.

[32] Aksentijevich I., Putnam C. D., Remmers E. F. (2007). The clinical continuum of cryopyrinopathies: novel CIAS1 mutations in North American patients and a new cryopyrin model. *Arthritis and Rheumatism*.

[33] Hoffman H. M., Mueller J. L., Broide D. H., Wanderer A. A., Kolodner R. D. (2001). Mutation of a new gene encoding a putative pyrin-like protein causes familial cold autoinflammatory syndrome and Muckle-Wells syndrome. *Nature Genetics*.

[34] Verma D., Särndahl E., Andersson H. (2012). The Q705K polymorphism in NLRP3 is a gain-of-function alteration leading to excessive interleukin-1*β* and IL-18 production. *PLoS ONE*.

[35] Ito S., Hara Y., Kubota T. (2014). CARD8 is a negative regulator for NLRP3 inflammasome, but mutant NLRP3 in cryopyrin-associated periodic syndromes escapes the restriction. *Arthritis Research and Therapy*.

[36] Kubota K., Ohnishi H., Teramoto T. (2014). Clinical and genetic characterization of Japanese sporadic cases of periodic fever, aphthous stomatitis, pharyngitis and adenitis syndrome from a single medical center in Japan. *Journal of Clinical Immunology*.

[37] Dinarello C. A. (2005). Blocking IL-1 in systemic inflammation. *Journal of Experimental Medicine*.

[38] Galon J., Aksentijevich I., McDermott M. F., O'Shea J. J., Kastner D. L. (2000). TNFRSF1A mutations and autoinflammatory syndromes. *Current Opinion in Immunology*.

[39] Stojanov S., Kastner D. L. (2005). Familial autoinflammatory diseases: genetics, pathogenesis and treatment. *Current Opinion in Rheumatology*.

[40] Stojanov S., Hoffmann F., Kéry A. (2006). Cytokine profile in PFAPA syndrome suggests continuous inflammation and reduced anti-inflammatory response. *European Cytokine Network*.

[41] Førsvoll J., Kristoffersen E. K., Øymar K. (2013). Elevated levels of CXCL10 in the periodic fever, aphthous stomatitis, pharyngitis and cervical adenitis syndrome (PFAPA) during and between febrile episodes; an indication of a persistent activation of the innate immune system. *Pediatric Rheumatology*.

[42] Yamazaki T., Hokibara S., Shigemura T. (2014). Markedly elevated CD64 expressions on neutrophils and monocytes are useful for diagnosis of periodic fever, aphthous stomatitis, pharyngitis, and cervical adenitis (PFAPA) syndrome during flares. *Clinical Rheumatology*.

[43] Sundqvist M., Wekell P., Osla V. (2013). Increased intracellular oxygen radical production in neutrophils during febrile episodes of periodic fever, aphthous stomatitis, pharyngitis, and cervical adenitis syndrome. *Arthritis and Rheumatism*.

[44] Valenzuela P. M., Araya A., Pérez C. I. (2013). Profile of inflammatory mediators in tonsils of patients with periodic fever, aphthous stomatitis, pharyngitis, and cervical adenitis (PFAPA) syndrome. *Clinical Rheumatology*.

[45] Martinez F. D., Stern D. A., Wright A. L., Taussig L. M., Halonen M. (1998). Differential immune responses to acute lower respiratory illness in early life and subsequent development of persistent wheezing and asthma. *Journal of Allergy and Clinical Immunology*.

[46] Wibrow B. A., Ho K. M., Flexman J. P., Keil A. D., Kohrs D. L. (2011). Eosinopenia as a diagnostic marker of bloodstream infection in hospitalised paediatric and adult patients: a case-control study. *Anaesthesia and Intensive Care*.

[47] Petra D., Petra K., Michaela K. (2015). Polyclonal, newly derived T cells with low expression of inhibitory molecule PD-1 in tonsils define the phenotype of lymphocytes in children with Periodic Fever, Aphtous Stomatitis, Pharyngitis and Adenitis (PFAPA) syndrome. *Molecular Immunology*.

[48] Kroemer G., Hirsch F., González-García A., Martínez-A C. (1996). Differential involvement of Th1 and Th2 cytokines in autoimmune diseases. *Autoimmunity*.

[49] Street N. E., Mosmann T. R. (1991). Functional diversity of T lymphocytes due to secretion of different cytokine patterns. *The FASEB Journal*.

[50] Sahoo M., Ceballos-Olvera I., Del Barrio L., Re F. (2011). Role of the inflammasome, IL-1*β*, and IL-18 in bacterial infections. *TheScientificWorldJournal*.

